# Choledochoplasty by Vein Grafts in Iatrogenic Bile Duct Injuries

**DOI:** 10.1155/1992/75089

**Published:** 1992

**Authors:** Aws S. Salim

**Affiliations:** 1 University Department of Surgery The Medical City Baghdad Iraq; 2 The Department of Surgery Ward 6 Stobhill General Hospital Glasgow G21 3UW UK

## Abstract

The quality of immediate repair of common bile duct injuries with or without tissue loss occurring during elective cholecystectomy is crucial and maybe the sole factor behind future stricture formation with its considerable morbidity and mortality. Successful repair of iatrogenic common bile duct injuries has been achieved by immediate saphenous vein grafts in two patients with cystic duct avulsion, in one patient whose duct was split by a balloon catheter, and in one patient where a segment of the duct was resected. Follow-up for 5 years demonstrated that the grafting remained sound and produced no complications. Consequently, the immediate repair of iatrogenic bile duct injuries using vein grafts deserves consideration.


*HPB Surgery*, 1992, Vol. 5, pp. 195–202
Reprints available directly from the publisher
Photocopying permitted by license only

© 1992 Harwood Academic Publishers GmbH
Printed in the United Kingdom

### CHOLEDOCHOPLASTY BY VEIN GRAFTS IN IATROGENIC BILE DUCT INJURIES

AWS S. SALIM
*University Department of Surgery, The Medical City, Baghdad, Iraq*

*(Received 25 November 1991)*

The quality of immediate repair of common bile duct injuries with or without tissue loss occurring during elective cholecystectomy is crucial and maybe the sole factor behind future stricture formation with its considerable morbidity and mortality. Successful repair of iatrogenic common bile duct injuries has been achieved by immediate saphenous vein grafts in two patients with cystic duct avulsion, in one patient whose duct was split by a balloon catheter, and in one patient where a segment of the duct was resected. Follow-up for 5 years demonstrated that the grafting remained sound and produced no complications. Consequently, the immediate repair of iatrogenic bile duct injuries using vein grafts deserves consideration.

KEY WORDS: Iatrogenic, bile duct injuries, choledochoplasty, saphenous vein

### INTRODUCTION

Despite recent developments and advances in gallstone dissolution, cholecystectomy remains one of the most frequently performed abdominal operations[1,2]. Regardless of the level of experience and skill on the side of the surgeon and even with his greatest care iatrogenic injury to the bile ducts continues to be one of the complications of cholecystectomy that poses a formidable problem. Fortunately this complication is not common. Kune and Sali[2] in a review of the literature from centres in which surgery is usually performed by trained surgeons found that the incidence of bile duct injury is about 1 in 300–500 operations for gallstones. The incidence varies between 0.15 and 0.2 per cent in European countries[3]. Factors implicated in causing iatrogenic bile duct injuries during cholecystectomy are variations in normal anatomy, cholecystectomy more than 5 days following an attack of acute cholecystitis, and haemorrhage in the region of the porta hepatis[1]. Occasionally, the injury is recognized immediately at operation and reconstruction of a divided duct is carried out by an end-to-end repair, however the failure rate of such repairs remains in the region of 50 per cent of all cases[3,4,5]. Consequently, this leads to the development of bile duct strictures that may be extremely difficult to correct and are associated with high mortality[3,6,7]. On the other hand, ductal injuries with tissue loss are usually repaired by a choledochojejunal anastomosis or by a serosal patch. The management of iatrogenic bile duct strictures is a dynamic issue. Vein patches have been used in situations in which there is a short, easily

Address correspondence to: Aws S. Salim, The Department of Surgery, Ward 6, Stobhill General Hospital, Glasgow G21 3UW, UK

accessible common bile duct stricture. Michie and Gunn[9] carried out a direct suture of a common bile duct stricture and when they were unable to close the anterior wall, they inserted a vein patch. The patient was well two years later. Ellis and Hoile[10] reported two cases of successful vein patch repair of post-cholecystectomy common bile duct strictures with follow-up periods of 9 years and one year respectively. They suggested that progressive growth takes place in the duct wall in a circumferential manner with slow shrinkage and eventual absorption of the vein patch.

Despite these findings the use of veins for the immediate repair of iatrogenic bile duct injuries has been ignored. This report presents the results of such repairs carried out by the author, who had already successfully performed choledochoplasty by veins in experimental animals as part of a programme aiming at excisional surgery for benign and malignant common bile duct strictures in man, and who was, therefore, given permission by the Medical City authorities to repair bile duct injuries caused by others.

### PATIENTS AND METHODS

Two males (47 and 53 years old) and two females (42 and 57 years old) were admitted for an elective cholecystectomy. Apart from biliary colics, their past medical history was unremarkable. The two women and 47 year old man were slightly over-weight. In all cases, liver function tests were normal and ultrasonography showed thickening of the gallbladder wall and numerous stones within its lumen. The intra- and extra-hepatic ducts were not dilated and did not appear to contain any stones.

At induction of anaesthesia an intravenous dose of one gram ampicillin (Penbritin, Beecham Laboratories, Brentford, Middx, UK) and 80 mg gentamicin (Cidomycin, Roussel Laboratories, Uxbridge, Middx, UK) were given. Because of the bile duct injury, these antibiotics were administered intravenously (500 mg ampicillin every 6 hours and 40 mg gentamicin every 8 hours) for 2 days. Peroperative pneumatic compression leggings were used as prophylaxis against deep vein thrombosis. In all cases the abdomen was opened through a right paramedian incision, which gave adequate exposure of the gallbladder and extra-hepatic biliary tree, the gallbladder was aspirated and the bile sent for bacteriological examination, no T-tube drainage of the common duct was used unless stated otherwise, a Redivac suction drain placed against the gallbladder bed and common duct was used at the end of each operation, and no peritoneal lavage was employed. The terminal part of the left long saphenous vein was used for all the repairs. This vein, which was not varicose in any patient, was obtained through a verticle 10 cm incision situated below the inguinal ligament over its terminal part after carefully ligating all its tributaries with 5/0 Polydioxanone (PDS, Ethicon, Edinburgh, UK) taking care not to compromise its lumen.

### *1. The 53-year Old Man*

Marked adhesions between the gallbladder and omentum were divided before the former was aspirated. The cystic duct was dissected and its junction with the common duct displayed. Then its gallbladder end was ligated. The cystic artery was divided between ligatures. A peroperative cholangiogram by direct needling of the common duct with a 22-gauge needle showed no filling defect suggesting the presence of stones. When traction was applied onto the cystic duct to reveal its junction with the common duct, it avulsed producing loss of a round area of common duct tissue approximately one cm in diameter. Direct closure of such a defect would have jeopardised the duct's lumen, therefore, repair using a saphenous free graft patch was planned. The gallbladder was dissected out retrogradely. Then 5 cm of the long saphenous vein was obtained. The lumen was flushed with warm saline, then the vein was opened longitudinally and the desired size of patch cut off. This patch was stitched in a reversed position with its endothelium inwards to the edges of the duct's defect using continuous 5/0 PDS (Ethicon Ltd., Edinburgh, UK). The repair was inspected for 15 minutes during which time no leakage occurred.

### 2. *The 57-year Old Woman*

After aspirating the gallbladder, a forceps was applied to Hartmann's pouch, however with the first traction to display the cystic duct, the gallbladder was detached revealing a 1.5 cm in diameter stone situated at the junction of the common and cystic ducts. The latter was so stretched that it made the gallbladder neck appear to be adherent directly to the common duct. The cystic artery was ligated, the common duct aspirated, the gallbladder dissected out retrogradely, then a Fogarty balloon catheter was used to check that the common duct contained no stones. The defect in the duct was approximately 1.5 cm in diameter and was repaired using a saphenous vein patch as described above. No leakage occurred when the repair was inspected for 15 minutes.

### 3. *The 42-year Old Woman*

After dividing adhesions to the gallbladder and aspirating it, the cystic duct was ligated. The cystic artery was divided between ligatures, then a perioperative cholangiogram by direct needling (22-gauge) of the common duct was performed. This revealed two stones which were not detected by ultrasonography. The gallbladder was dissected out retrogradely, then the common duct was opened between stay sutures and the stones removed using a Fogarty balloon catheter. When this catheter was used to check the hepatic portion of the biliary tree and when inflating the balloon while pulling downstream along the common hepatic duct, the latter split longitudinally producing a 2 cm tear in direct continuity with the choledochotomy. Thus, the biliary duct had an opening about 4.5 cm long and it was thought that direct repair may induce excessive fibrosis which may in the future compromise the lumen. A decision was, therefore, taken in favour of using an elliptical saphenous vein patch to keep the duct edges apart and to ensure a wide lumen. This patch was prepared as described earlier and cut to a length matching the bile duct opening and being widest in the middle (about one cm) and tapering towards each end. It was stitched in a reversed direction with its endothelium inwards to the edges of the choledochotomy using continuous 5/0 PDS. The integrity of the anastomosis was inspected for 15 minutes and no leakage occurred.

*4. The 47-year Old Man*

No adhesions were found between the gallbladder and other intraperitoneal structures. After aspirating the gallbladder and ligating the cystic duct and dividing the cystic artery, an operative cholangiogram was performed and this showed no abnormality. When the apparent junction between the cystic and common ducts was ligated and divided, it was realised that the common duct was excessively mobile and, therefore, pulled out of its place forming a U-shaped segment in continuity with a short wide cystic duct. This U-shaped portion was ligated and divided causing loss of one cm of common duct. The duct's ends were caught in bulldog clips and trimmed with a bevel. The gallbladder was dissected out retrogradely. Five cm of the long saphenous vein were excised, flushed with warm saline, divided into two equal lengths which were then split longitudinally and anastomosed together using continuous 5/0 PDS to form one wide tube. This anastomosis was initially performed on each side at the middle only and after trimming the ends with bevels matching those of the common duct and providing a length of venous tube that can bridge the gap without tension, the anastomosis was completed. The bulldog clips were then removed and the venous tube was anastomosed in a reversed manner to the ends of the common duct using continuous 5/0 PDS. A T-tube was inserted into the common duct above the anastomosis. Inspection for 15 minutes showed no leakage of bile at the anastomotic lines. A T-tube cholangiogram undertaken on the 14th post-operative day without prior clamping revealed a completely intact common duct without any compromise of its lumen. The T-tube was then removed without any complications.

None of these cases had excessive inflammatory adhesions making the cholecystectomy difficult and none had any significant anatomical anomalies that could be held responsible for injury development.

### RESULTS

All the patients made an uneventful recovery from their operations and none of them had any significant pains that could be attributed to the choledochoplasty. They all comfortably tolerated free fluids by mouth then solid food without any complications. Pyrexia (apart from the initial post-operative reactionary rise in temperature) or significant leucocytosis was not observed in any patient. After an initial small volume collection in all the suction drain bottles, no further volumes were drained and there was no sign of biliary leakage at any of the repairs. Twenty four hours post-operatively, the suction drain bottles were changed and the new ones remained empty over the following 24 hours when the drains were removed. An ultrasonography on the third post-operative day showed no evidence of intraperitoneal collections and no dilatation of the biliary tree. In all cases, liver function tests including BSP excretion performed on the 4th post-operative day were of values within normal limits.

The patient with a T-tube was discharged home on the 16th post-operative day whereas all the remaining patients were allowed home between the 6th and 7th post-operative days.

These patients were followed up at 6 weeks, 6 months, 1 year, 2 years, 3 years, 4 years and 5 years post-operatively. During each visit liver function tests including

BSP excretion, right hypochondrial ultrasonography, and an intravenous cholangiogram were carried out. The repair remained sound in all patients and produced no strictures or any compromise of the duct's lumen.

### DISCUSSION

Despite knowledge of the circumstances under which iatrogenic bile duct injuries occur during elective cholecystectomy[1,3], it seems that such injuries will continue to appear even in the hands of the most experienced surgeons[8]. Most of the operations in which the injuries occur are complicated by inflammatory tissue alterations rendering the dissection hazardous or by significant haemorrhage in the region of the porta hepatis, however, in some of these operations failure to recognize anatomical anomalies remains the sole factor responsible for injury development[1,8]. In the present series none of these factors could be incriminated in the mechanism of the bile duct injuries, thus emphasizing the importance of the highest degree of vigilance on the part of the operator even when the field does not appear to harbour any cause for alarm. Simple tractions on the cystic duct can cause avulsion injuries or distort the junction with the common duct to such an extent that a mobile common duct can be pulled laterally and so put out of shape that it could be mistaken for being a continuation of the cystic duct. Iatrogenic bile duct injuries caused by instruments should be easily avoidable with continued training and extra care. The bile duct injury caused by the Fogarty balloon might have been avoided if the balloon was inflated several times before using it to acquire an idea about balloon size responses. On the other hand, impaction of a large stone at the junction of the cystic and common ducts attenuates the tissues at this junction by chronic inflammation and causes loss of their elasticity by the consequent fibrosis to such a degree that the slightest pull on the junction can produce an avulsion injury with tissue loss. It must be stressed, however, that those in training who perform biliary surgery must always be supervised by experienced senior surgeons who have the knowledge to recognize the first step on the road to disaster. The injuries presented were not inflicted by the author and it is, therefore, unknown whether excessive traction was applied to the cystic duct or whether some of the injuries presented could have been avoided with more experience and extra care.

Debate continues about the place of peroperative cholangiography in aiding the prevention of iatrogenic bile duct injuries. Collins and Gorey[1] reported that the value of this investigation cannot be underestimated in providing a comprehensive picture of the biliary tree. Mathisen *et al.*[8], however, argue that cholangiography is not the most important factor in prevention of bile duct injuries and should not be a guideline for dissection, but remain an examination to disclose disease, an opinion supported by the data of the present report.

Surgical trauma to the bile duct is a serious complication for the patient and is a real problem for the surgeon. Occasionally the injury is recognized immediately at operation and repaired, however Bismuth[3] reported that failure of this repair and the incidence of subsequent stricture formation can be as high as 50%. Such strictures may be extremely difficult to correct and are associated with high mortality[1,3,5–8]. It, thus, appears that success of the initial repair is crucial in preventing, or at least minimizing, future complications of iatrogenic bile duct injuries. Therefore, in the cases presented vein grafts were employed to enable successful repair of common duct injuries with or without tissue loss. It was felt that T-tube drainage is unnecessary for the repair unless it was carried out for complete transection of the duct. These repairs were successfully performed without any morbidity or mortality.

The importance of follow-up after primary repair of bile duct injuries cannot be overemphasised. Such follow-up is vital for the early detection of strictures and prevention of complications like infection and secondary biliary cirrhosis. In the cases studied, follow-up and investigations aimed at examining the integrity of the common duct were carried out for 5 years. Bile duct strictures did not occur in any case thereby supporting the use of vein grafts for choledochoplasty. Current methods for the immediate repair of bile duct injuries are governed by the type of injury incurred. It is customary to undertake an end-to-end anastomosis for complete transection and to carry out serosal patching or choledochojejunostomy when the injury has caused tissue loss[1,2,8]. These methods are either associated with a disheartening morbidity[3] or cause unnecessary disruption of the gastrointestinal tract. It is, therefore, hoped that the use of veins for the repair would offer a suitable alternative that helps to minimize the complications of other methods.

The argument that the use of veins means subjecting the patient to a second simultaneous operative procedure which may itself produce some morbidity like wound infection besides prolonging the operation time, should not be a deterrent against exploring the full potential of choledochoplasty by veins because there is much to be gained if the incidence of strictures occurring after ductal repair could be reduced.

In conclusion, immediate repair of common bile duct injuries using vein grafts may have advantages over other forms of this repair and deserves consideration.

*Acknowledgements*

I am grateful to Dr S.H. Alwash for giving me the opportunity to undertake this work at the Medical City and I thank Mrs Jutta Gaskill for her skilful secretarial work.

*References*

1. Collins, P.G. and Gorey, T.F. (1984) Iatrogenic biliary stricture: presentation and management. *Br. J. Surg.*, **71**, 980--982
2. Kune, G.A. and Sali, A. (1980) *The Practice of Biliary Surgery*, 2nd Edition pp. 192–239. Oxford: Blackwell
3. Bismuth, H. (1982) Post-operative strictures of the bile duct. In: Blumgart, L.H., Ed., *The Biliary Tract*, pp. 209–218. Edinburgh: Churchill-Livingstone
4. Way, L. and Dunphy, J.E. (1972) Biliary stricture.*Am. J. Surg.*, **124**, 287–295
5. Cattell, R.B. and Braasch, J.W. (1959) General considerations in the management of benign strictures of the bile duct. *New Engl. J. Med.*, **261**, 929–933
6. Warren, K.W. and Jefferson, M.F. (1973) Prevention and repair of strictures of the extrahepatic bile duct. *Surg. Clin. North Am.*, **53**, 1169–1175
7. Blumgart, L.H., Kelley, C.J. and Benjamin, I.S. (1984) Benign bile duct stricture following cholecystectomy. Critical factors in management. *Br. J. Surg.*, **71**, 836–838
8. Mathisen, O., Bergan, A. and Flatmark, A. (1987) Iatrogenic bile duct injuries. *World J. Surg.*, **11**, 392–397

9. Michie, W. and Gunn, A. (1964) Bile duct injuries: a new suggestion for their repair. *Br. J. Surg.*, **51**, 96–100
10. Ellis, H. and Hoile, R.W. (1980) Vein patch repair of the common bile duct. *J. Roy. Soc. Med.*, **73**, 635–637

*(Accepted by S. Bengmark 27 November 1991)*

### INVITED COMMENTARY

This report by Salim is of value because it extends the experience with the use of vein grafts in reconstruction of the biliary tree with four cases, in whom no untoward effects of the use of vein grafts was seen. With the advent of the great popularity of laparoscopic cholecystectomy with its attendant much higher risk of bile duct damage, any advance in the care of patients with biliary tract trauma is most welcome. This technique, of course is borrowed from the basic manoeuver of vein graft angioplasty, where the graft is thought to be nourished directly by the intraluminal blood. In the case of its use in the biliary tract, the source of its oxygen supply and nutrients is unknown.

In the four cases reported, there is an excellent description of the technique and a very good follow-up. However, in none of the cases is the percentage of circumference which is damaged described or is there an estimation of the original diameter of the bile duct. This information would be valuable in assessing the results of these procedures. In addition it would be valuable to have cholangiographic follow-up on the contours of the grafted ducts over the time periods mentioned.

There are four types of acute injury to the bile ducts: crushing injuries, avulsions, ligatures, and segmental excisions. Venous grafts are probably of use only in avulsions, where significant amounts of the circumference of the bile ducts have been removed. Grafts in these cases take the place of serosal patches, which have never been extensively used in biliary surgery. Ligature injuries to the bile ducts are probably best handled by de-ligating the duct and then placement of a T-tube through the damaged portion of duct. These tubes should be left in for from three to six months. Crushing injuries depend on the width of the crush for the most optimal reconstruction. With an extremely narrow crush, perhaps circumferential suturing and stenting with a T tube for three to six months would be adequate. For wide crushings, and also segmental excisions, trimming of the damaged portion of the duct with an anastomosis to preferably the jejunum or duodenum is probably better than the use of a segmental vein graft since there is only one anastomosis at risk with the former, but with the vein grafts there are two.

The author mentions that there is considerable morbidity and mortality associated with the repair of biliary tract injuries. More recent reports[1,2,3,4,5] indicate that outside of the requirement of perhaps another operative procedure, the mortality is extremely low (0–4%), the chance of success is quite satisfactory (67–86%) and the morbidity is not too bad. However, the author correctly points out that it is far better to avoid further surgery by accomplishing the repair at the time of the cholecystectomy. One study which is not mentioned records the results of repairs of biliary injury at the time of cholecystectomy which indicates that the mortality is 0% but the recurrence rate is 46% if hepatico-jejunostomy is used[6].

### *References*

1. Braasch, J.W., Bolton, J.S. and Rossi R.L. (1981) A technique of biliary tract reconstruction with complete follow-up in 44 consecutive cases. *Ann. Surg.,* **194**, 635–638
2. Castrini, G. and Pappalardo, G. (1981) Iatrogenic strictures of the bile ducts: our experience with 66 cases. *World J. Surg.*, **5**, 753–758
3. Genest, J.F., Nanos, E., Grundfest-Broniatowski, S., Vogt, D. and Hermann, R.E. (1986) Benign biliary strictures: an analytic review (1970–1984). *Surgery,* **99**, 409–413
4. Pellegrini,C.A., Thomas, M.J. and Way, L.M. (1984) Recurrent biliary strictures: patterns of recurrence and outcome of surgical therapy. *Am. J. Surg.,* **147**, 175–180
5. Pitt, H.A., Miyamoto, T., Parapatis, S.K., Tompkins, R.K. and Longmire, W.P. Jr. (1982) Factors influencing outcome in patients with postoperative biliary strictures. *Am. J. Surg.,* **144**, 14–21
6. Andred-Sandberg, A., Johansson, S. and Bengmark, S. (1985) Accidental lesions of the common bile duct at cholecystectomy. II: Results of treatment. *Ann. Surg.,* **201**, 452–455

John W. Braasch

